# Notes of Surgical Cases in the Buffalo General Hospital

**Published:** 1862-05

**Authors:** J. F. Miner


BUFFALO

### Medical and Surgical Journal

AND REPORTER.

VOL. I. MAY, 1862. NO. 10.

### ORIGINAL COMMUNICATIONS.

ART. I.—NOTES OF SURGICAL CASES IN THE BUFFALO GENERAL HOSPITAL. *Impassable Stricture of Urethra—Perineal Fistula—Operation; Croup—Tracheotomy—Death; Depressed Fracture of Skull: By* J. F. MINER, M. D.

*Case* 1*st—Stricture, Perineal Fistula, Operation.*—J. Haunstein, German, aged 48 years. Admitted to the Surgical Ward January 8th, 1861. Ten years since, while in Germany, received injury, by being crushed in some heavy machinery, through the pelvis, since which time has had more or less pain and difficulty in urinating. Within the last three years has been under the care of various surgeons for treatment of stricture of the urethra, and one year since was operated upon for the relief of the stricture and fistula, which had now been present for a year or more, and through which the urine mostly escaped. This operation was attended with partial temporary relief, but the urethra was again closed in about four months, and at the time of admission the urine passed wholly through a fistulous opening near the point of the ischium. The urine was mixed with great quantities of mucus, which was secreted abundantly by the lining of the track, and by the mucus surfaces of the urethra and bladder. Two other openings were also found communicating with the urethra, one in front of the scrotum by direct passage, and one posterior to it by a sinuous canal. The patient was emaciated, exsanguine, of sallow complexion, very weak, with a peculiar expression of pain and anxiety. The urine was constantly escaping in small quantity, while as often as every twenty or thirty minutes it was voided in much greater amount with pain and bearing down effort. Relief of the bladder was thus found to occupy considerable time, the pain resembling more that of labor than anything else to which it could be compared. All effort to introduce a dilating instrument of any kind having failed, and the necessities of the case being urgent, assisted by my colleagues. Drs. Eastman and Wilcox and medical students, a grooved staff was introduced to the point of stricture, and an incision, as for lithotomy, made down upon the point. No director or other guide could be passed through the strictured portion, and the dissection was extended in the supposed direction of the urethra, extending the whole length of the membraneous portion, which was found completely closed and impervious, and reaching the prostatic portion, which was considerably dilated. This part of the operation, termed by authors, the "most difficult, embarrassing and uncertain operation attempted by surgeons,'' was at length accomplished, with much less delay than had been anticipated, and we passed a metalic catheter through the urethra into the bladder. The parts healed kindly over the catheter, which was retained constantly, being occasionally removed, cleaned, and re-introduced. The fistulous passages united, except the larger one through which the urine had so long made its escape, that the lining had taken on the characters of mucus membrane, in so much, that, though no urine escaped, still it did not unite.

One year after the operation the patient still introduces the catheter so frequently or retains it so constantly, as but rarely to void urine without it. This grows out of an unconquerable fear that if the instrument is dispensed with, the passage will again contract. Various caustic and stimulating injections were used with the view of exciting adhesive action in the fistule, but with very little success, though it seemed to cause contraction and some improvement, so that it is productive of no great inconvenience. The general health is fully restored, and the hardest labor is now performed. The urine is clear and healthy; and the relief afforded by the treatment is as perfect and satisfactory as could possibly be expected.

Cases demanding such operation are fortunately very rare; few are presented to the surgeon where properly conducted efforts for diletation are unsuccessful; indeed the greatest care should be taken, that every other resource is exhausted before an operation is decided upon. There are many features in this case of exceeding practical interest; perhaps no one case could illustrate more important points, but the one for especial remembrance is, the contraction of the passage after the first operation, showing the necessity of retaining or of introducing the catheter for a long time in order to prevent such accident. The failure of the first operation was probably due to this neglect.

*Case 2d—Croup, Tracheotomy, Death.*—A little girl six and a half years old, of previous good health, was noticed early Wednesday morning, Feb'y 8, 1861, to be very hoarse, and to breathe with some sufficulty. At ten o'clock she was admitted to the medical ward, under Dr. Wyckoff. with the following symptoms:—Skin moist; pulse 120 per minute; speaks only in whisper; dyspnœa very great; slight cough; general redness of the fauces, with no appearance of false membrane. Dr. W. prescribed sulphate of zinc and ipecac as an emetic, which operated very soon, affording not the slightest relief. I was now invited to see the child with Dr. Wyckoff. We regarded the case as exceedingly unpromising, and as affording very little if any prospect of relief from medical treatment. Tracheotomy was proposed, as offering the only remaining hope, slight indeed, and hardly to be entertained. We advised pulvis ipecac et opii, and sulph. quiniæ, two grains each every four hours, and appointed four o'clock P. M., for again visiting the child. The morning was the true time to operate, if at all, as every hour was diminishing our chances of success, yet we reluctantly determined to delay for the purpose of obtaining the advice and assistance of our colleagues.

*Four o'clock P. M.*—Respiration more difficult, pulse more rapid and feeble; surface livid, and all the indications of approaching death from asphyxia. About seven o'clock, the child being under the influence of chloroform, assisted by several members of the Hospital staff and medical students, I proceeded to the operation of tracheotomy. The struggles of the child, constant rapid motion of the trachea, profuse hæmorrhage, the effects of the chloroform, and other circumstances greatly embarrassed the operation, and when at length the tube was introduced, it immediately filled with a membraneous product, three or four inches in length, completely filling the tube and necessitating its removal. Again it was replaced, and we had the pleasure of seeing our little patient breathe with ease and comfort. On Thursday morning we found our patient very comfortable, breathing with great ease, taking water, beef essence, &c., and appearing cheerful and hopeful; hopes were now entertained of her recovery. In the afternoon, respiration became more frequent, pulse more feeble, and the rapidly increasing prostration, with evidences of bronchial inflammation, pointed too certainly to a fatal termination. She died thirty hours after the operation, and the morbid specimens afford points of great interest and importance. The inflammatory exudation was so profuse and abundant as to completely fill the trachea, as if moulded into it. Below the point where the tube was introduced, there was observed a more recent formation, or product. The first was removed at the time of the operation, but it was afterwards renewed and extended into the minutest bronchial tubes, which we were able to trace. This exudation was more extensive than I have ever seen. The rapidity with which the effusion takes place, and its extension over large surfaces, are characteristics of the croupous exudation.

Prof. Rochester has examined this product under the microscope, and reports that it is the common croupous exudation.

*Case 3d—Depressed Fracture of the Skull.*—Thomas Ley, aged 26 years, admitted December 5th. He had fallen from the cars on the Central railroad, while in full motion. The frontal bone was fractured and driven down upon the brain with scalp, hair, dirt, etc., etc. He was insensible, breathing slowly and stertorously; had convulsions before my arrival at hospital, and was supposed to be dying; had vomited the contents of stomach with large quantities of blood. By using the trephine we made a clean opening in the skull, and carefully removed from the brain structure, large quantities of bone, dirt, &c., with at least one ounce of brain matter, which was so bruised as to be taken off with the removal of foreign matter. During this operation he had a severe convulsion, and I delayed the work, thinking him about dead. The fracture extended to the longitudinal sinus on the one side, below to the orbital plate, and we had the skull removed for a space of about three inches by two. Most of the fragments of bone and foreign matter were removed from over the orbital plate. Hæmorrhage was profuse, but abated somewhat upon the application of water dressing, and a light bandage. His respiration gradually became more easy and natural, and he appeared to rally a little, growing warm, and showing approaching reaction. The next day he told us his name; the day after he gave his father's name and place, since which time he has answered all questions slowly by speech, or by very deliberate motion or sign. All the functions of the brain and organic life are properly and naturally performed without apparent loss of power, while yet quite a large portion of brain substance is wanting. It is very remarkable that this injury did not produce immediate death from concussion, causing suspension of all nervous influence, but having escaped this, the sources of danger are yet very numerous, a few of which I will mention. Compression would seem sufficient to certainly cause death in a very short time. Hæmorrhage, from which he also escaped, though it was profuse, and continued in some degree for several days. Loss of brain substance, which, as I have said, seems in this case to be no great loss after all. Fungus, from which, at one time, it seemed certain he would suffer, disappeared upon careful compression. Inflammation with effusion or disorganization is also to be greatly feared. The very abundant discharge, depression of nervous energy, disinclination for food, and other influences greatly increase the danger of exhaustion. I have attempted only to give a brief account of the nature and extent of this injury, interesting mainly as showing the remarkable powers of nature.

*May* 10, 1861.—Five months after the accident, while working at some wood in the yard, this patient fell suddenly and died. He has not been well since the injury, though he has been at his accustomed labor of chopping wood, but has never enjoyed his former health and strength. Disease of the brain, or rupture of some of the vessels weakened by the injury, was probably the cause of death. Post mortem examination was refused.

